# Sputum Glucose and Glycemic Control in Cystic Fibrosis-Related Diabetes: A Cross-Sectional Study

**DOI:** 10.1371/journal.pone.0119938

**Published:** 2015-03-24

**Authors:** Lindsey Van Sambeek, Elise S. Cowley, Dianne K. Newman, Roberta Kato

**Affiliations:** 1 California Institute of Technology, Pasadena, California, United States of America; 2 Children’s Hospital Los Angeles, Pediatric Pulmonology, Los Angeles, California, United States of America; University of Athens Medical School, GREECE

## Abstract

Cystic fibrosis-related diabetes affects up to half of cystic fibrosis patients and is associated with increased mortality and more frequent pulmonary exacerbations. However, it is unclear to what degree good glycemic control might mitigate these risks and clinical outcomes have not previously been studied in relation to glucose from the lower airways, the site of infection and CF disease progression. We initially hypothesized that diabetic cystic fibrosis patients with glycosylated hemoglobin (HbA_1c_) > 6.5% have worse pulmonary function, longer and more frequent exacerbations and also higher sputum glucose levels than patients with HbA_1c_ ≤ 6.5% or cystic fibrosis patients without diabetes. To test this, we analyzed spontaneously expectorated sputum samples from 88 cystic fibrosis patients. The median sputum glucose concentration was 0.70 mM (mean, 4.75 mM; range, 0-64.6 mM). Sputum glucose was not correlated with age, sex, body mass index, diabetes diagnosis, glycemic control, exacerbation frequency or length, or pulmonary function. Surprisingly, sputum glucose was highest in subjects with normal glucose tolerance, suggesting the dynamics of glycemic control, sputum glucose and pulmonary infections are more complex than previously thought. Two-year mean HbA_1c_ was positively correlated with the length of exacerbation admission (p < 0.01), and negatively correlated with measures of pulmonary function (p < 0.01). While total number of hospitalizations for exacerbations were not significantly different, subjects with an HbA_1c_ > 6.5% were hospitalized on average 6 days longer than those with HbA_1c_ ≤ 6.5% (p < 0.01). Current clinical care guidelines for cystic fibrosis-related diabetes target HbA_1c_ ≤ 7% to limit long-term microvascular damage, but more stringent glycemic control (HbA_1c_ ≤ 6.5%) may further reduce the short-term pulmonary complications.

## Introduction

Proper blood glucose management of cystic fibrosis-related diabetes (CFRD) adds a substantial burden to patients who already spend hours every day managing pulmonary, GI, and other complications of cystic fibrosis (CF). Microvascular complications of CFRD typically arise more than 10 years after diagnosis [1], when CF patients are approaching their median life expectancy in their mid-30s [2]. In addition to potential long-term complications, evidence suggests that CFRD reduces pulmonary function [3] and increases the frequency and likelihood of treatment failure [4, 5] in pulmonary exacerbations (PEx), the primary cause of mortality in CF [6, 7]. It is unclear by what mechanism CFRD negatively impacts pulmonary health and if stringent glycemic control would reduce these effects. It is frequently hypothesized that CFRD increases sputum glucose (SG), which could support airway pathogens and contribute to PEx [8, 9].

Work by Brennan et al. [10] and Baker et al. [11] indicates that hyperglycemia causes an increase in glucose concentrations in nasal secretions and breath condensates of CFRD patients, and these have been used as proxies for airway glucose in lower airways. However, no studies have examined the effects of sputum glucose on pulmonary health, even though sputum directly represents the conditions of the small airways frequently infected and damaged in CF. To better characterize the environment of the lower airways in CF, we measured SG from adult and pediatric CF patients at baseline and during PEx. We also conducted a two-year chart review to provide context for SG measurements and to investigate if stringent glycemic control in CFRD was associated with better pulmonary health. We found that while sputum glucose was quite low and not associated with CFRD, average glycosylated hemoglobin (HbA_1c_) ≤ 6.5% was associated with improved health outcomes for CFRD patients.

## Methods

### Ethics statement: patient recruitment and chart review

CF patients from Children’s Hospital Los Angeles and Keck Hospital of USC were consented and assented in writing in accordance with the Institutional Review Board at Children’s Hospital Los Angeles and the University of Southern California Health Science Institutional Review Board, IRB# CCI-13–00211 and HS-14–00068, respectively. The entire study was reviewed and approved by the Institutional Review Board at Children’s Hospital Los Angeles. Informed consent from a parent or legal guardian was obtained for patients < 18 years old, and assent of patients 13–18 years old was also obtained. Inclusion criteria were ≥ 4 years old, CF diagnosis, and the ability to spontaneously produce sputum. A retrospective chart review of subjects’ health records from January 2012—December 2013 was performed to obtain age, ethnicity (self-reported as white, Hispanic, black or other), body mass index (BMI), history of PEx, CFRD status, and sputum culture and blood test results. Oral glucose tolerance tests determined glucose tolerance according to Cystic Fibrosis Foundation and American Diabetes Association guidelines [12]. For data analysis, CFRD with and without fasting hyperglycemia were considered as one group. BMI is used as an indicator of nutrition in CF patients and this was considered in the analyses. We did not have enough information to control for socioeconomic status or environmental exposures.

### Sputum acquisition and preparation

Sputum samples were processed as described by Hunter et al. [13]. Briefly, samples were flash-frozen in liquid nitrogen immediately upon expectoration and stored at -80°C. Upon thawing, samples were briefly centrifuged at 8,000 x *g*, 1 min to separate sputum from saliva. The mass of sputum samples was measured, and an equal mass of 50 mM 4-(2-hydroxyethyl)-1-piperazineethanesulfonic acid (HEPES) buffer was added. This was homogenized by repeatedly passing through a #16 needle, then centrifuged at 8,000 x *g*, 10 minutes. The supernatant was filtered on a 0.2 μm-pore-size column at 10,000 x *g*, 20 minutes.

### Sputum glucose measurement

Filtered sputum supernatant was tested with a colorimetric glucose hexokinase assay (Sigma-Aldrich, GAHK20) calibrated for glucose concentrations 0–22.2 mM on a 96-well plate reader (BioTek). Samples were measured in triplicate and the average compared to a standard glucose curve on each plate. Resulting values were doubled to account for the HEPES dilution described in section 2.2. Samples that initially tested above the calibrated range of the assay and from which sufficient supernatant remained were diluted five-fold with Millipore water and re-tested.

### Statistical analysis

Calculations of mean and median, standard deviation and error, t-tests and regression analyses were performed using JMP Pro 11. P-values < 0.05 were considered statistically significant.

## Results

### Study participant demographics

Eighty-eight subjects with CF were enrolled. Their demographic information is described in Table 1 and also available in the Supplementary Information Data Tables (S1 Dataset and S2 Dataset). Most of the subjects were white or Hispanic (84%), and 45% had abnormal glucose tolerance. On average, the subjects were young adults of BMI within one SD of CFF recommendations [14]. Mean forced expiratory volume in one second (FEV_1_) score is consistent with moderate disease severity.

**Table 1:** Study participant demographic information, n = 88.

Characteristic	
**Age** (years), mean (SD)	24.3 (10.0)
**Male / Female**, n (%)	49 (56) / 39 (44)
**Ethnicity**, n (%)	
White	50 (57)
Hispanic	34 (39)
Black / other	4 (5)
**BMI, male** (kg/M^2^), mean (SD)	21.2 (3.3)
**BMI, female** (kg/M^2^), mean (SD)	20.8 (3.7)
**FEV_1_** (% predicted), mean (SD)	62.7 (26.6)
**FEF25–75** (% predicted), mean (SD)	43.4 (30.7)

### Sputum glucose distribution

**Fig 1:**
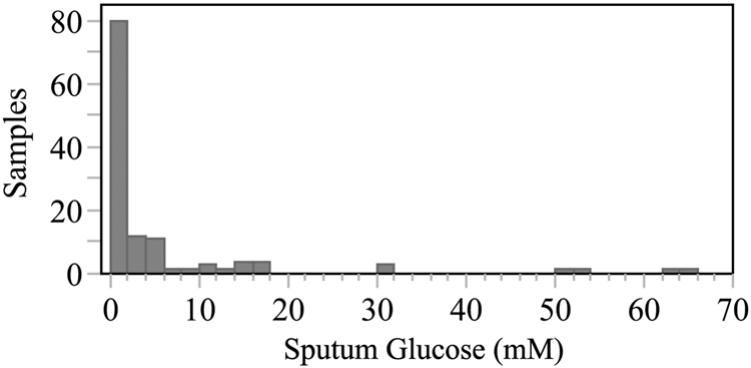
Distribution of sputum glucose concentration in 117 samples. Sputum glucose (SG) was much lower than previously reported, with 34% reading < 0.01 mM, and 55% of samples reading < 1.0 mM. The distribution of SG was highly skewed: the median concentration was 0.7 mM (IQR, 0–3.5 mM) versus the mean of 4.75 mM (SD 11.4 mM).

To characterize SG, we tested sputum samples from the subject cohort described in Table 1, with multiple samples from 21 subjects. Sputum glucose was < 0.01 mM in 34% and <1.0 mM in 55% of sputum samples (40 and 64, respectively, out of 117 samples, see Fig. 1). The distribution of SG was highly skewed: the median concentration was 0.7 mM (IQR, 0–3.5 mM) versus the mean of 4.75 mM (SD 11.4 mM). There were no clear trends in SG over time from individual patients who provided multiple samples.

### SG and clinical parameters

**Fig 2:**
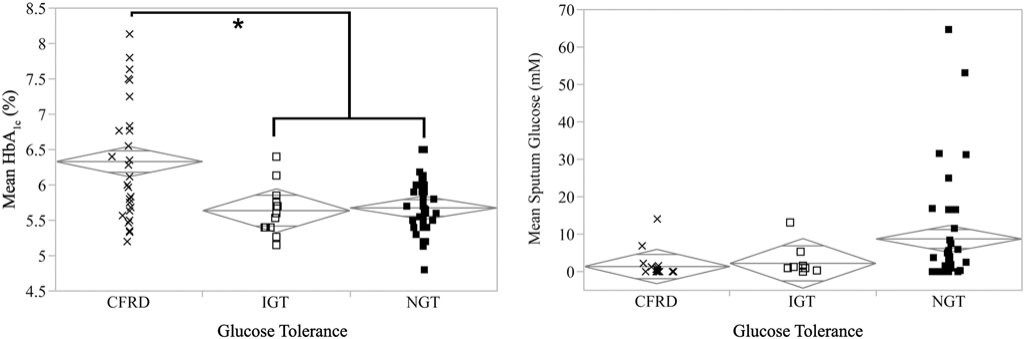
Comparisons of HbA_1c_ and SG by glucose tolerance. CFRD subjects had significantly higher HbA_1c_ than subjects with impaired (IGT) or normal glucose tolerance (NGT, left panel), while NGT subjects had significantly higher average sputum glucose than IGT or CFRD subjects (right panel). Diamonds are centered on each category’s mean and the vertical distance represents the 95% confidence interval.

To determine if SG values were correlated with glucose tolerance, we compared data from our chart review with SG values. Two-year mean HbA_1c_ was highest in subjects with CFRD, as expected (see Fig. 2). However, SG was not increased in CFRD, but rather was significantly higher in those with normal glucose tolerance (NGT). Further bivariate analyses with other clinical values did not show significant correlations (see Table 2). There was a statistically significant positive correlation between SG and forced expiratory flow in the 25–75% of exhalation (FEF 25–75%), but given the nearly flat trendline and low R^2^, this is likely not clinically significant.

**Table 2:** R^2^ and p-values from correlations between SG and clinical parameters.

Clinical parameter	R^2^	(p value)
**Age** (years)	0.01	(0.99)
**Sex** (ANOVA)	<0.01	(0.55)
**Ethnicity** (ANOVA)	<0.01	(0.55)
**BMI** (kg/M^2^)	<0.01	(0.94)
**FEV1** (% predicted)	<0.01	(0.65)
**FEF 25–75%** (% predicted)	0.09	(<0.01)
**CFRD diagnosis** (ANOVA)	0.09	<0.01
**HbA_1c_** (%)		
mean (2012–2013)	<0.01	(0.54)
at sputum collection	0.02	(0.34)
**PEx status**, at sputum		
collection (ANOVA)	0.05	(0.07)
**Total PEx** (2012–2013)	0.01	(0.29)
**Mean PEx admission length**		
(2012–2013)	<0.01	(0.24)

### CFRD and pulmonary health

Because SG was not correlated with glucose tolerance, we analyzed PEx hospital admission history over the prior two years to determine if CFRD subjects in this cohort had clinically significant pulmonary health differences compared to CF subjects without diabetes.

CFRD subjects had twice as many pulmonary exacerbations during the previous two years as subjects with impaired (IGT) or normal (NGT) glucose tolerance (mean, 5.00 vs 2.27 and 2.59, SE = 0.56, p < 0.01). There was no difference in PEx admission length in CFRD, IGT or NGT subjects (13.2, 11.2, 9.8, p = 0.14).

Given that CFRD subjects had more PEx but not increased SG, we next reviewed clinical data to determine if glycemic control, as indicated by HbA_1c_, was associated with differences in pulmonary health. We stratified subjects using the two-year mean HbA_1c_, defining stringent control as HbA_1c_ ≤ 6.5% and poor control as HbA_1c_ > 6.5%. These two groups had no statistically significant differences in age, sex, or BMI, although subjects with poor control had been diagnosed with CFRD for longer, as shown in Table 3.

**Table 3:** Clinical parameters of CFRD subjects.

Clinical parameter	HbA1c > 6.5%	HbA1c ≤ 6.5%	p value
**Male / Female**, n	4 / 6	9 / 8	
**Ethnicity**, n			
White / Hispanic / Black and other	8 / 1 / 1	6 / 11 / 0	
**Age** (years), mean (SD)	30.6 (11.9)	25.8 (10.8)	0.15
**BMI** (kg/M^2^), mean (SD)	22.5 (4.7)	21.6 (4.4)	0.33
**CFRD duration** (years), mean (SD)	15.3 (9.3)	6.1 (8.1)	0.01
**Two-year mean HbA1c** (%), mean (SD)	7.3 (0.5)	5.8 (0.4)	<0.01
**SG** (mM), mean (SD)	0.70 (0.90)	1.7 (3.9)	0.18
**PEx total**, mean (SD)	4.6 (3.0)	5.3 (3.4)	0.31
**PEx admission** length (days), mean (SD)	15.1 (6.2)	11.9 (5.4)	0.10
**FEV_1_** (% predicted), mean (SD)	40.4 (14.5)	58.4 (18.2)	0.03
**FEF25–75%** (% predicted), mean (SD)	15.2 (8.7)	36.5 (22.9)	0.01

**Fig 3:**
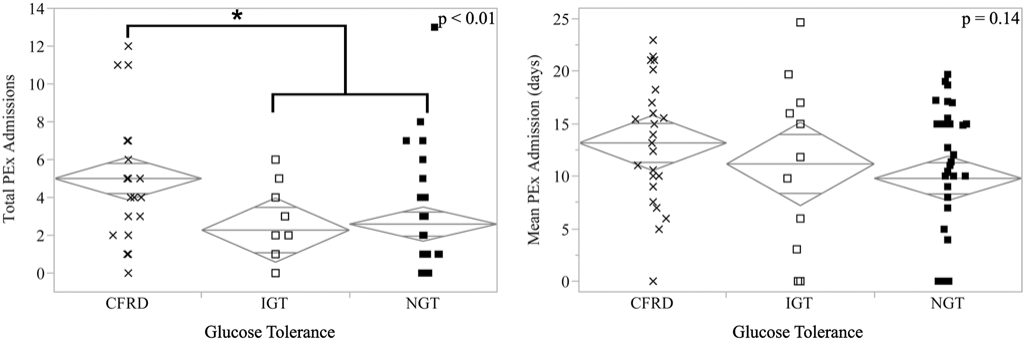
Glucose tolerance and two-year pulmonary exacerbation history. CFRD subjects had approximately twice as many pulmonary exacerbations as subjects with impaired (IGT) or normal glucose tolerance (NGT, left panel). The average length of admission was not significantly different between these groups (right panel). Diamonds are centered on each category’s mean and the vertical distance represents the 95% confidence interval.

Analysis of the entire study population revealed that those with a two-year average HbA_1c_ > 6.5% were admitted for 6 days longer than those with a two-year average HbA_1c_ ≤ 6.5% (mean: 16.1 vs 10.3, p <0.01, see Fig. 3) and scored lower measures of pulmonary function, FEV_1_ (37 vs 67% predicted, p < 0.01) and on forced expiratory flow in the middle 50% of the patient’s exhaled volume (FEF 25–75%, 14 vs 49% predicted, p < 0.01).

**Fig 4:**
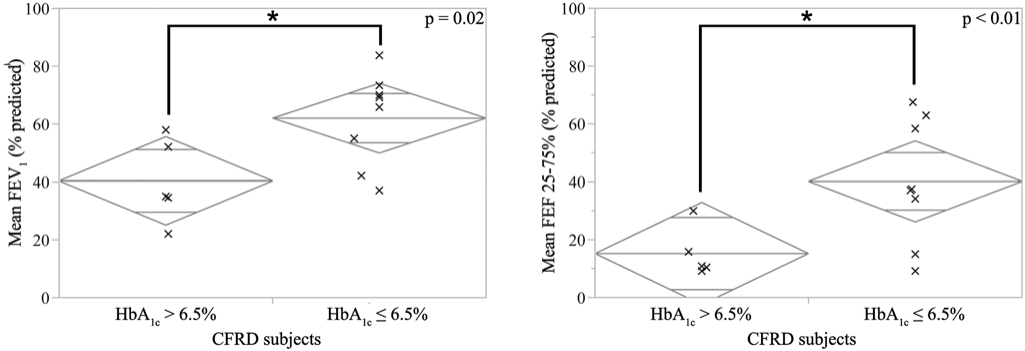
Mean pulmonary function test results by glycemic control in CFRD. CFRD subjects with HbA_1c_ > 6.5% had significantly reduced average scores of forced expiratory volume in one second (FEV_1_, left panel) and forced expiratory flow in the 25–75% of the patient’s exhaled volume (FEF25–75%, right panel) compared to CFRD subjects with HbA_1c_ ≤ 6.5%. Diamonds are centered on each category’s mean and the vertical distance represents the 95% confidence interval.

Within CFRD subjects, those with poor glycemic control had significantly reduced average FEV_1_ and FEF25–75% scores. These findings suggest that glycemic control is correlated with reduced morbidity (see Fig. 4). Duration of diabetes, while higher in CFRD patients with poor glycemic control, was not directly correlated with PEx admission length or frequency or mean FEV_1_ or FEF25–75%.

## Discussion

We sought to determine if CFRD worsens health through an increase in SG, which could foster bacterial growth and worsen pulmonary infections. However, our study found SG was highest in subjects with NGT as compared to IGT or CFRD. Overall, SG concentration was usually quite low in comparison with previously reported SG values in adult CF patients. CFRD subjects had higher morbidity than CF subjects without diabetes and poor glycemic control was associated with longer PEx hospitalizations and worse pulmonary function.

Glucose in CF sputum was previously measured in just four samples from nonexacerbating adults by Palmer et al. [15], reporting a mean concentration of 3.2 mM. This finding has been widely used in synthetic CF sputum medium for *in vitro* models of CF airways. While the mean SG found here was similar to Palmer’s (4.8 vs 3.2 mM), our average is strongly skewed by a few outlying high values. It is likely more physiologically relevant to describe SG by the median of 0.7 mM, or even 0 mM, as was often the case. Our results have the advantage of a much larger sample size (117 vs 4) from pediatric and adult subjects, with the health records to put these findings in context. The lack of relationship between increased SG and CFRD diagnosis suggests the increased morbidity in CFRD is independent of SG.

The link between CFRD and worsening pulmonary function has been described previously. CFRD subjects have shown a greater rate in decline of FEV_1_ than NGT subjects [3], with the most rapid decline correlating with the lowest endogenous insulin production [16]. Sims et al [17] also showed a significant decrease in lung function in female CFRD subjects when compared to NGT controls. Despite the tendency for worse health outcomes once CFRD has been diagnosed, it is encouraging that our results correlated lower HbA_1c_ with shorter PEx and better pulmonary function. While HbA_1c_ is impacted by many factors besides glycemic control [18], this suggests subjects may be able to minimize the short-term pulmonary damage from CFRD with management of blood glucose—in addition to reducing long-term microvascular complications of CFRD [19]. However, patients and physicians must weigh the benefits of tight glycemic control against the increased risk of hypoglycemia. It is also encouraging that duration of diabetes, a factor completely beyond the control of subjects and their physicians, was not directly correlated with more or longer PEx or worsening pulmonary function.

One possible mechanism of CFRD’s negative impact on pulmonary health is insulinopenia: insulin is an anabolic hormone important in signaling tissue growth and repair [16] and acts on airway epithelial and smooth muscle cells [20], so a shortage of insulin could contribute to worsening pulmonary function in CFRD. One study supported this hypothesis by reversing the decline in pulmonary function of CFRD subjects with one year of insulin therapy [21]. Alternatively, Waugh et al. [22] has suggested that as CFRD indicates endocrine pancreatic insufficiency, it may also mark exocrine pancreatic insufficiency and worse malabsorption. The resulting undernutrition would limit nutritional resources and thus also the body’s ability to repair lung tissue from chronic and acute infections.

The surprising correlation between higher SG and NGT is counterintuitive. We initially hypothesized that higher serum glucose levels in CFRD would correspond to higher sputum glucose levels; however, there was a trend in the opposite direction. Insulin has been shown to modulate transporters’ activity in lung epithelial cells [20], and it is conceivable that increased insulin (associated with NGT) triggers greater flux of glucose across the airway epithelium, resulting in higher SG. Alternately, microorganisms in the CF lung may affect SG and overall pulmonary function. Just as infection patterns elsewhere in the body are different for diabetics than those with NGT, the lung microbiome may also be altered in the context of CFRD. For instance, if CFRD subjects have higher levels of glucose-utilizing organisms, these pathogens may take up glucose preferentially, leaving comparatively little glucose in the extracellular milieu of sputum. In the future, comparative microbiome community sequencing and transcriptomic analyses could help test this hypothesis. However, one should note that the HbA_1c_ range in these subjects was relatively narrow (5.2–8.1%), and these findings may not apply to patients with higher HbA_1c_ levels.

Certain limitations to our findings should be considered. First, we have more sputum samples from subjects who were more frequently in clinic and admitted to the hospital. Thus, our samples may over-represent exacerbating conditions. For instance, only 48% (56/117) of the sputum samples were from well subjects, although most of our subjects are well more often than not. A second limitation is that the samples are not uniformly from a fasting state versus specific post-prandial time point which could acutely affect SG levels and might account for the few outlying high values. Lastly, sputum samples are subject to contamination from food or drink residues in the oropharynx as the sputum was expectorated, which could artificially increase the tested SG concentration.

To better describe the conditions that affect SG and determine its peak concentration, future work could focus on patients with continuous glucose monitors, or collecting multiple samples over the course of an oral glucose tolerance test, when a spike in blood glucose is induced and carefully followed. Glucose concentration in nasal secretions and breath condensates follows blood glucose on a similarly rapid time scale [10, 11, 23], but the dynamics of lower airway SG are unknown. The potential for SG as a predictor of an oncoming PEx also remains untested. Additionally, the interactions between CF lung flora and SG are unexplored. For example, how do microorganisms contribute to or deplete SG, and what biological effects does SG have on bacterial growth rates, pathogenicity, and antibiotic resistance?

In conclusion, this work has shown that SG is frequently very low in CF subjects, but may increase dramatically for unknown reasons. The pathophysiology of how CFRD negatively impacts pulmonary health is evidently more complicated than a simple change in SG; however, lower HbA_1c_, which generally indicates better glycemic control, is associated with shorter PEx admissions and better pulmonary function. Current recommendations suggest a treatment goal of HbA_1c_ ≤ 7% for CFRD patients to reduce long-term microvascular complications [19], but a more stringent goal of HbA_1c_ ≤ 6.5% may also reduce short-term pulmonary complications and improve lung function.

## Supporting Information

S1 Dataset Data table 1. (CSV)

S2 Dataset Data table 2. (CSV)
